# Oral vaccination of foxes and raccoon dogs against rabies with the 3rd generation oral rabies virus vaccine, SPBN GASGAS, in Finland

**DOI:** 10.1186/s13028-021-00605-y

**Published:** 2021-10-13

**Authors:** Ad Vos, Tiina Nokireki, Marja Isomursu, Tuija Gadd, Ferenc Kovacs

**Affiliations:** 1 Ceva Innovation Center GmbH, Am Pharmapark, 06861 Dessau-Rosslau, Germany; 2 Virology Unit, Finnish Food Authority, Mustialankatu 3, 00790 Helsinki, Finland; 3 Veterinary Bacteriology and Pathology Unit, Finnish Food Authority, Elektroniikkatie 3, 90590 Oulu, Finland

**Keywords:** Rabies, Bait uptake, SAD B19, Seroconversion

## Abstract

**Background:**

To prevent re-emergence of wildlife-mediated rabies in Finland, oral rabies vaccine baits are distributed every year during autumn in southern Finland in a vaccination zone bordering Russia. Recently, Finland introduced a 3rd generation oral rabies virus vaccine bait. By analysing bait uptake and seroconversion in red foxes and raccoon dogs, the field efficacy of this new vaccine strain, SPBN GASGAS, was compared with the originally used highly efficacious 1st generation vaccine SAD B19.

**Results:**

Overall, 74.6% and 53.9% of the animals submitted from the vaccination area after the campaigns (2017–2019) tested positive for the presence of the bait marker and anti-rabiesvirus antibodies, respectively. No significant difference was observed between years, species and vaccine.

**Conclusions:**

The field performance of the highly attenuated 3rd generation oral rabies vaccine, SPBN GASGAS, in terms of bait uptake and seroconversion was similar to the 1st generation vaccine, SAD B19, and therefore offers a suitable alternative.

## Background

The last rabies outbreak in Finland occurred between April 1988 and February 1989. Interestingly, most cases were not observed in the known European reservoir species, the red fox (*Vulpes vulpes*), but predominantly in raccoon dogs (*Nyctereutes procyonoides*) [1]. This invasive species was first observed in Finland in the 1930s and after an initial phase of establishment its population increased greatly from the end of the 1960s onwards [2, 3]. In the years following the rabies outbreak in Finland, rabies cases in raccoon dogs were reported from many other European countries as well [4]. The epidemic in Finland was successfully controlled with oral vaccination whereby vaccine baits containing the 1st generation modified live rabies virus vaccine, SAD B19, were distributed in the infected and surrounding risk areas bordering Russia [5]. Subsequently, a vaccination corridor in this area was established and baits have been distributed annually to prevent spread of rabies ever since. Although highly efficacious, SAD B19 and other 1st generation vaccines are associated with certain safety issues such as residual pathogenicity and vaccine associated rabies cases have been reported from several countries in different animal species using this type of vaccine [6–10]. Hence, upon the development of more attenuated vaccines, vaccine baits containing 2nd and more recently a 3rd generation oral rabies vaccine virus were distributed in Finland. Due to non-availability of both the latter types of vaccine in 2017, the original 1st generation vaccine SAD B19 used to eliminate the outbreak at the end of 1980s was distributed once more. This offered the unique opportunity to compare the field performance of the highly attenuated 3rd generation vaccine used since 2018 with the highly efficacious 1st generation vaccine during the previous campaign in 2017 as both vaccines are filled in the same type of sachet and subsequently incorporated in an identical fish product bait matrix. The results of this comparison are presented in this study.

## Methods

### Vaccine baits

During the campaigns two different commercially available oral rabies vaccine baits were used; Fuchsoral in 2017 and Rabitec in 2018 and 2019. Fuchsoral contains the 1st generation oral rabies virus vaccine SAD B19 that has been used in many European countries since 1983 [11, 12]. SAD B19, is an attenuated rabies virus developed by conventional in vitro serial passaging of the original SAD Bern oral rabies vaccine strain [13]. Rabitec contains the 3rd generation rabies virus vaccine SPBN GASGAS, a genetically engineered vaccine based on a cDNA clone (SAD L16) of SAD B19. To eliminate the residual pathogenicity observed in adult mice after intracerebral inoculation with its parental strain SAD B19 and to circumvent a potential compensatory mutation all three nucleotides at amino acid position 333 and 194 of the glycoprotein gene have been altered, respectively. Also, SPBN GASGAS lacks the pseudogene (ψ) [14–16]. Furthermore, to enhance the safety profile of the vaccine virus an additional, identical modified glycoprotein was inserted [17]. The vaccine was filled in a PVC-blister covered with aluminium foil, subsequently the blister was incorporated in a bait matrix consisting of fish products and vegetable fats. Furthermore, the bait matrix contained 150 mg tetracycline (TC) as bait marker substance.

### Vaccination campaigns

The vaccination campaigns were carried out once a year in the autumn; from mid-September till early November (Table 1). The baits (n  =  180,000) were distributed in Finland along its south-eastern border towards Russia in a zone 20–40 km wide from Ilomantsi to Pyhtää. The total surface area of the aerial distribution is around 12,000 km^2^. Bait density was 14–15 baits/km^2^ and baits were exclusively distributed by fixed wing airplanes and flight lines (parallel) were 1000 m apart. The location of every bait drop was recorded.

**Table 1:** The distribution period of the vaccine baits and vaccine strain used for the three vaccination campaigns in Finland, 2017–2019

Year	Start	End	Vaccine strain
2017	Sept. 29th	Nov. 7th	SAD B19 (Fuchsoral)
2018	Sept. 15th	Oct. 14th	SPBN GASGAS (Rabitec)
2019	Sept 14th	Oct. 30th	SPBN GASGAS (Rabitec)

### Laboratory investigations

Foxes and raccoon dogs were collected all year round as part of hunting activities in cooperation with the Finnish Wildlife Agency and local hunters and hunters’ associations for post-campaign monitoring purposes (“control animals”). Fox hunting is carried out predominantly for recreational purposes and predator control. The aim was to get four foxes or raccoon dogs per 100 km^2^ of baited area, so 360 samples in total per year as baits were not distributed in certain areas within the vaccination area (e.g., water surfaces, inhabited areas). The Finnish Food Authority set a target number of samples for each hunting association in the vaccination area. The samples were sent to the Finnish Food Authority’s laboratory by the hunters. The hunters’ associations received a bounty for each animal they delivered.

For statistical analysis, only the animals submitted 1 month after the last day of the campaign till the end of April the following year were included. This endpoint was selected because of the young animals emerging from their den; these animals did not have access to the vaccine baits distributed the previous autumn. Control animals submitted during ongoing vaccination campaigns were removed from the data set as it was not possible to determine if the observed presence of TC or antibodies was a result of the ongoing or previous campaign. As the animals also need several weeks to develop detectable levels of antibodies, animals submitted during the first month after the end of the campaign were also not included.

### Vaccine virus detection

Brain samples were taken from all animals submitted for rabies diagnostic, including the control animals, and examined for the presence of rabies virus antigen using a direct fluorescent antibody test (FAT) [18]. To confirm or rule out that presence of rabies virus antigen in FAT-positive samples was due to vaccine virus, if needed, the virus was subsequently isolated by cell culture (Mouse Neuroblastoma Cells) and sequenced (PCR).

### Bait marker detection

For detection of the TC marker, a half of the lower jaw was used. A ca. 0.2 mm slice was sawed from behind the canine tooth so that both jawbone and the cross-section of the root of the canine tooth were included in the sample slice. Two or three slices per individual were prepared. Slices were mounted in glycerol and the presence of fluorescent TC in bone and/or teeth was examined in ultraviolet light at 20× and 100× magnification.

### Antibody detection

Seroconversion was analyzed by a commercial antibody ELISA kit BioPro according to the instruction from the manufacturer (BioPro Rabies ELISA, Czech Republic). Sera collected from the thoracic cavity were considered seropositive for RABV-antibodies if they showed a percentage of inhibition compared to the negative serum  >  40% in ELISA.

### Age and sex determination

Age class (juvenile  <  1 year or adult  ≥  1 year) and sex was determined at necropsy. Age determination was based on general development and teeth development and wear. In winter and spring, tooth sections for TC detection were used as additional tools for age class determination by examining pulp cavity width. Also, the presence of placental scars was used for confirmation of age (≥  1 year old) in females.

### Statistics

A multiple logistic regression analysis (MLR) was performed for bait uptake and seroconversion. For bait uptake (TC positive or negative), it was determined if age (adult/juvenile), sex, species (raccoon dog/red fox) and vaccine (Fuchsoral/Rabitec) had an influence on bait uptake. As variables ‘year’ and ‘vaccine’ matched perfectly, they cannot be incorporated together in the MLR-model (2017—Fuchsoral, 2018 and 2019—Rabitec). Hence, it was decided to omit variable ‘year’. A similar MLR-analysis for seroconversion was performed. Here the variable ‘bait uptake’ (TC positive / negative) was included. The Chi^2^-test was used for univariate data analysis. GraphPad Prism 9.0 (GraphPad Software Inc., San Diego, CA, USA) was used for statistical analysis.

## Results

After applying the omission criteria described above, data from 407 control animals were available for analysis: 314 (77.1%) raccoon dogs and 93 red foxes (22.9%).

Overall, 74.6% and 53.9% of the animals tested positive for the presence of the TC bait marker and antibodies against rabies virus, respectively (Tables 2, 3). 23.5% (71/226) of the animals showed evidence of bait consumption but did not have antibodies against rabies virus and 2.3% (7/76) of the animals had detectable levels of antibodies but no evidence of bait uptake (Table 4). All animals submitted for rabies diagnostic including control animals tested negative for the presence of rabies virus antigen in the brain.

**Table 2:** Bait uptake, presence of bait marker—tetracycline, according to year (2017–2019), species (red fox and raccoon dog) and vaccine (SAD B19, SPBN GASGAS)

Campaign	Positive	
	n/N	%
Raccoon dog		
2017	62/83	74.7
2018	102/37	74.5
2019	42/58	72.4
Fox		
2017	33/41	80.5
2018	28/39	71.8
2019	3/4	75.0
Year		
2017	95/124	76.6
2018	130/176	73.9
2019	45/62	72.6
Vaccine		
Fuchsoral	95/124	76.6
Rabitec	175/238	71.5
Species		
Raccoon dog	206/278	74.1
Fox	64/84	76.2
Total	270/362	74.6

*n* number of animals that tested positive for bait marker; *N* total number of animals examined for bait marker

**Table 3:** Seroconversion rate based on the presence of antibodies (ELISA), according to year (2017–2019) and species (red fox and raccoon dog)

Campaign	Positive	
	n/N	%
Raccoon dog		
2017	44/71	62.0
2018	70/132	53.0
2019	35/60	58.3
Fox		
2017	14/33	42.4
2018	18/38	47.4
2019	6/13	46.2
Year		
2017	58/104	55.8
2018	88/170	51.8
2019	41/73	56.2
Vaccine		
Fuchsoral	58/104	55.8
Rabitec	129/243	53.1
Species		
Raccoon dog	149/263	56.7
Fox	38/84	45.2
Total	187/347	53.9

*n* number of animals that tested positive for the presence of antibodies (inhibition  >  40%); *N* total number of animals examined for the presence of antibodies

**Table 4:** Number and percentage (in parenthesis) of animals per species and year that tested positive and/or negative for the presence of the bait marker (TC^+^/TC^−^) and antibodies (Ab^+^/Ab^−^)

	TC^+^/Ab^+^	TC^+^/Ab^−^	TC^−^/Ab^+^	TC^−^/Ab^−^
Species				
Red fox	34 (45.3)	21 (28.0)	0 (0)	20 (26.7)
Raccoon dog	121 (53.3)	50 (22.0)	7 (3.1)	49 (21.6)
Year				
2017	56 (54.4)	22 (21.4)	1 (1.0)	24 (23.3)
2018	80 (50.0)	40 (25.0)	2 (1.3)	38 (23.8)
2019	19 (48.7)	9 (23.1)	4 (10.3)	7 (17.9)
Vaccine				
Fuchsoral	56 (54.4)	22 (21.4)	1 (1.0)	24 (23.3)
Rabitec	99 (49.8)	49 (24.6)	6 (3.0)	45 (22.6)
Total	155 (51.3)	71 (23.5)	7 (2.3)	69 (22.8)

No significant difference between years (Chi^2^  =  0.45, df  =  2, P  =  0.80), species (Chi^2^  =  0.15, df  =  1, P  =  0.70) and vaccines (Chi^2^  =  0.41, df  =  1, P  =  0.52) in bait uptake was detected. Also, no significant difference between years (Chi^2^  =  0.61, df  =  2, P  =  0.73), species (Chi^2^  =  3.34, df  =  1, P  =  0.07) and vaccines (Chi^2^  =  0.21, df  =  1, P  =  0.46) in seroconversion rate was observed.

## Regional differences

**Fig. 1:**
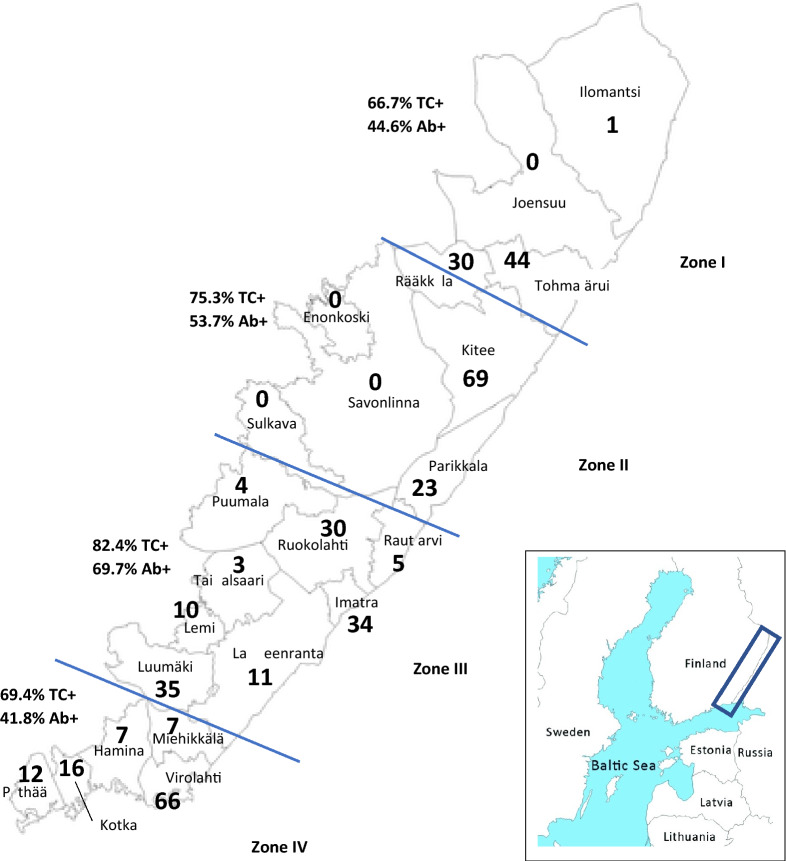
Percentage of animals that tested positive for the bait marker tetracycline (TC^+^) and for antibodies against rabies (Ab^+^) in the four zones (Zone I, II, III, IV) of the vaccination belt in Finland bordering Russia during the 2017–2019 campaigns. Also, the number of animals per municipality in the vaccination corridor used for the analysis are shown

The number of animals submitted per municipality in the vaccination area together with the bait uptake and seroconversion rate of the 4 regions are shown in Fig. 1; Table 5. No regional differences in bait uptake between the 4 regions were observed: Chi^2^  =  7.192, df  =  3, P  =  0.066. However, a significant difference in seroconversion rate was found between the regions, due to a higher seroconversion in Region III; Chi^2^  =  18.64, df  =  3, P  =  0.003.

**Table 5:** Number of animals included per campaign year and species for every municipality in the vaccination area and the bait uptake and seroconversion rate per region

Region	Municipality	2017	2018	2019	Red fox	Raccoon dog	TC^+^ (%)	Ab^+^ (%)
I	Ilomantsi	–	1	–	1	–		
	Joensuu	–	–	–	–	–		
	Rääkkylä	11	13	6	10	20		
	Tohmajärvi	10	16	18	13	31		
	Subtotal	21	30	24	24	51	66.7	44.6
II	Kitee	24	33	12	19	50		
	Enonkoski	0	0	0	0	0		
	Savonlinna	0	0	0	0	0		
	Sulkava	0	0	0	0	0		
	Parikkala	4	15	4	2	21		
	Subtotal	28	48	16	21	71	75.3	53.7
III	Puumala	2	2	0	1	3		
	Ruokolahti	16	14	0	4	26		
	Rautjärvi	1	3	1	1	4		
	Imatra	5	16	13	3	31		
	Taipalsaari	0	1	2	0	3		
	Lemi	6	4	0	0	10		
	Lappeenranta	4	7	0	5	6		
	Luumäki	4	31	0	5	30		
	Subtotal	38	78	16	19	113	82.4	69.7
IV	Miehikkälä	1	3	3	1	6		
	Virolahti	22	13	31	13	53		
	Hamina	4	3	0	3	4		
	Kotka	3	8	5	3	13		
	Pyhtää	8	3	1	8	4		
	Subtotal	38	30	40	28	80	69.4	41.8
	Total	125	186	96	92	315	74.6	53.9

## Multiple logistic regression

The results (odds ratio) of the MLR-analysis are summarized in Table 6. Sex and species did not have an influence on bait uptake in contrast to age. For seroconversion, only bait uptake had a highly significant impact; as bait uptake is a prerequisite for seroconversion this was to be expected. The other variables had no significant impact on seroconversion. Although the odds ratio for Rabitec was 1.3, indicating a positive effect on seroconversion, it was not significant.

**Table 6:** Parameter estimates from the multiple logistic regression model associated with bait uptake and seroconversion in foxes and raccoon dogs after oral rabies vaccination campaigns in Finland

Parameter	Odds ratio	95% CI	Reference setting
Seroconversion			
Tetracycline—positive/negative	0.049	0.019–0.108	Positive
Age—juvenile/adult	1.017	0.576–1.784	Juvenile
Sex—female/male	1.066	0.617–1.840	Female
Species—red fox/raccoon dog	0.657	0.348–1.243	Raccoon dog
Vaccine—Fuchsoral/Rabitec	1.309	0.560–3.222	Fuchsoral
Bait uptake			
Age—juvenile/adult	0.433	0.254–0.725	Juvenile
Sex—female/male	1.015	0.612–1.688	Female
Species—red fox/raccoon dog	1.009	0.535–1.849	Raccoon dog

## Discussion

Overall, 74.6% of the animals tested positive for the bait marker; 74.1% and 76.2% of the raccoon dogs and foxes, respectively. Meanwhile, seroconversion rates were lower; 56.7% in raccoon dogs and 45.2% in foxes. Roughly, a 20% (raccoon dog) to 30% (fox) difference between bait uptake and seroconversion rate was found. This discrepancy is most often a result of bait handling as some animals may simply have eaten the bait mass without puncturing the capsule or most of the released vaccine was spilled during bait consumption. For induction of an immune response, the vaccine must be released in the oral cavity and enter the body via the mucous membrane or tonsils [19]. In Lithuania, a country with comparable epidemiological settings as Finland, a similar difference was found as 78.1% and 52.2% of the red foxes tested positive for presence of the bait marker and antibodies between 2006 and 2013, respectively [20]. However, in more recent years (2010–2019), the reported seroconversion rate dropped in Lithuania to 31.9% and 34.1% for foxes and raccoon dogs, respectively [21]. In some countries, extremely large differences between bait uptake and seroconversion have been observed. In Estonia, another country in the Baltic region, 43% and 36% of the foxes and raccoon dogs examined between 2006 and 2010 tested positive for the bait marker but did not have detectable antibodies [22]. In Serbia, the difference between bait uptake (67.6%) and seroconversion (23.3%) in foxes was also extremely large (2011–2014) [23].

Many factors can influence bait uptake and subsequently seroconversion. For example, the composition and ingredients of the bait matrix used in Finland has changed. In the original bait matrix used during the 1988 and 1989 campaigns, terrestrial animal products were used. However, it was replaced by fish products due to the BSE-crisis. Also, other slight adaptations in the proportions of other components were made to increase the melting point of the bait matrix. Hence, these changes may have influence bait attractiveness and consumption and possibly explain the higher bait uptake rates during the campaigns at the end of the 1980s. For example, after the first campaign in September 1988, 78.6% and 87.5% of raccoon dogs and foxes tested positive for the bait marker, respectively [1]. Bait uptake rate as determined by the presence of the bait marker by the target species is complex and depends on many factors. First of all, the number of baits distributed determines the availability to the target species, but this number is relative to the population density not only of the target species but also of the non-target species that also consume baits distributed. Bait depletion by non-target species, like the wild boar (*Sus scrofa*), as determined by the presence of antibodies against rabies, indicate that bait competition by this animal species can be substantial [24, 25]. In recent years, a clearly visible warning sticker has been added on the outside of the bait matrix with the aim of reducing potential human contact with the vaccine. The originally inconspicuous baits can now easily be detected and it is assumed that the warning sticker most likely increases bait depletion by well-known bait competitors like corvids that forage by visual clues. Furthermore, the warning sticker could also have a negative effect on bait acceptance and handling by the target species. Additionally, the temporal and spatial availability of more preferred food sources can affect bait uptake [26]. The bait distribution system can also have a profound effect on bait availability and consequently bait uptake by the target species. Background prevalence of tetracycline in foxes and raccoon dogs due to environmental contamination with this widely used antibiotic is feasible. However, it was shown that background levels of tetracycline were extremely low in targeted mesocarnivores in Canada [27]. Finally, the composition of the animals in the sample can play a role. During the present study, the age of the animals had a significant effect on the presence of the bait marker. As observed in other studies [22], the proportion of juveniles testing positive for the bait marker was lower than for adults. This can be explained by the fact that adults in contrast to juveniles have experienced more than one vaccination campaign and thus have a higher probability of having encountered and consumed baits.

The seroconversion rate is affected by additional factors. For example, it is influenced by the assay used and the cut-off set for sero-positivity [28, 29]. In Finland during the campaigns in 1988 and 1989 a virus neutralization assay was used for detection of virus neutralizing antibodies. Nowadays, seroconversion is determined here by the presence of virus binding antibodies using an ELISA. Hence, comparing seroconversion rates between the first and more recent campaigns has little value. The quality of the vaccine can have a direct influence on the immune response. Low seroconversion rates have been linked with baits distributed that contained vaccines with titres below the minimum effective dose [30]. This does not only have to be related to a low-quality vaccine batch but can also be a result of an inadequate cold chain. Climatic conditions can also impact the quality of the vaccine baits distributed; when exposed to high temperatures a significant reduction in vaccine titre and even inactivation can occur [31, 32]. Low temperatures can have a negative impact on the seroconversion rate as well. When baits are dropped during periods with sub-zero (<  0 °C) temperatures, the liquid vaccine may become solid frozen and when an animal perforates the blister with its teeth, the vaccine is not released properly resulting in a failed vaccination attempt. However, this is not applicable to Finland as baits are distributed only during September–October when temperatures are generally above 0 °C. The presence of maternal antibodies can interfere with active vaccination in young animals during late spring oral vaccination campaigns. Thus, these young animals can locate and consume baits but will develop a low or undetectable immune response [33]. When recombinant oral rabies vaccines are used, pre-existing immunity against the vector virus can negatively impact the immune response against rabies [34–36]. Therefore, it is not surprising that due to the many factors impacting bait uptake and especially seroconversion observed rates show such a wide variation throughout Europe in time and space; reported seroconversion rates varied from 17.8% in Bulgaria to 94.0% in Poland [37, 38]. Hence, great care should be taken when the results of post-campaign monitoring like bait uptake and seroconversion rate from different areas and time periods are compared with each other. However, using the same distribution protocol (spatial and temporal), vaccination area, and vaccine bait matrix, no difference in bait uptake and seroconversion was found between the 1st (SAD B19) and 3rd (SPBN GASGAS) generation vaccine. Despite the high efficaciousness of 1st generation oral rabies vaccines and their undisputable contribution to the elimination of fox-mediated rabies in Europe [12], they have a major disadvantage; a low residual pathogenicity. This may be acceptable during the initial elimination phase as numbers of vaccine-associated cases reported are low and without epidemiological relevance [39] but becomes more controversial once the disease has been eliminated and baits are distributed in rabies-free areas to prevent re-infection from neighboring areas as in Finland.

## Conclusions

Rabitec containing the 3rd generation oral rabies vaccine SPBN GASGAS offers a safe and efficacious alternative to the previous 1st and 2nd generation oral rabies vaccines used in Finland.

## Data Availability

The datasets used and/or analysed during the current study are available from the corresponding author on reasonable request.
